# RNAseq of Gingival Fibroblasts Exposed to PRF Membrane Lysates and PRF Serum

**DOI:** 10.3390/cells13151308

**Published:** 2024-08-05

**Authors:** Atefe Imani, Layla Panahipour, Hannes Kühtreiber, Michael Mildner, Reinhard Gruber

**Affiliations:** 1 Department of Oral Biology, University Clinic of Dentistry, Medical University of Vienna, Sensengasse 2a, 1090 Vienna, Austria; dr_a_imani@hotmail.com (A.I.); layla.panahipour@meduniwien.ac.at (L.P.); 2 Department of Dermatology, Medical University of Vienna, 1090 Vienna, Austria; n01471143@students.meduniwien.ac.at (H.K.); michael.mildner@meduniwien.ac.at (M.M.); 3 Applied Immunology Laboratory, Department of Thoracic Surgery, Medical University of Vienna, 1090 Vienna, Austria; 4 Department of Periodontology, School of Dental Medicine, University of Bern, 3010 Bern, Switzerland; 5 Austrian Cluster for Tissue Regeneration, 1200 Vienna, Austria

**Keywords:** platelet-rich fibrin (PRF), RNAseq, membrane, serum, gingival fibroblasts, chemokines, inflammation, in vitro

## Abstract

Platelet-rich fibrin (PRF) is prepared by spontaneous coagulation of fractionated blood. When squeezed between two plates, PRF is separated into solid PRF membranes and a liquid exudate, the PRF serum. The question arises regarding how much the overall activity remains in the PRF membranes and what is discarded into the PRF serum. To this end, we have exposed gingival fibroblasts to lysates prepared from PRF membranes and PRF serum, followed by bulk RNA sequencing. A total of 268 up- and 136 down-regulated genes in gingival fibroblasts exposed to PRF membrane lysates were significantly regulated under the premise of a minimum log2 with 2.5-fold change and a minus log10 significance level of two, respectively. PRF serum only caused 62 up- and 32 down-regulated genes under these conditions. Among the 46 commonly up-regulated genes were CXCL1, CXCL5, CXCL6, CXCL8, IL33, IL6, and PTGS2/COX2, stanniocalcin-1—all linked to an inflammatory response. PRF membrane lysates further increased chemokines CCL2, CCL7, CXCL2, CXCL3, and IL1R1, IL1RL1, and IL1RN, as well as the paracrine factors IL11, LIF, IGF1, BMP2, BMP6, FGF2, and CCN2/CTGF, and all hyaluronan synthases. On the other hand, PRF serum increased DKK1. The genes commonly down-regulated by PRF membrane lysates and PRF serum included interferon-induced protein with tetratricopeptide repeats (IFIT1, IFIT2, IFIT3) and odd-skipped-related transcription factors (OSR1 and OSR2), as well as FGF18 and GDF15, respectively. Taken together, PRF membrane lysates, compared to PRF serum, cause a more complex response in gingival fibroblasts, but each increased chemokine expression in gingival fibroblasts.

## 1. Introduction

Platelet-rich fibrin (PRF), the coagulated plasmatic fraction of blood that is enriched in platelets and leucocytes [1,2], has gained clinical relevance as an autologous fibrin-rich matrix supporting regeneration—a strategy that was initially established in the dental field [3,4] and has been applied to intra-oral bone regeneration [5], sinus floor augmentation [6], treatment of periodontal intrabony defects [7], and gingival recession [8]. PRF has also been recognized in other disciplines for its potential to support the healing of diabetic foot ulcers [9,10], for instance, as well as for applications in sports medicine, dermatology and cosmetology, gynecology and reproductive medicine, ophthalmology, and neurology [11]. Thus, there is a large spectrum of possible applications for PRF membranes in dentistry and other fields. When preparing PRF membranes, obtaining the coagulated fibrin-rich plasmatic fraction of fractionated blood requires squeezing out the respective PRF serum, also termed PRF exudate [12,13,14]. Thus, the PRF serum is devoid of the fibrin-rich matrix, blood cells, and related coagulation factors. The PRF serum is consequently discarded as a residual liquid. The PRF membrane, however, is a fibrin-rich matrix reinforced by the activated platelets [15] and is thus a clinically applicable temporary matrix with intrinsic bioactivity and favorable biomechanical properties [16]. The question arises as to what activity is lost to the PRF serum, in part because PRF serum is clinically used, for instance, as eye drops in the management of cornea and ocular diseases [17,18].

PRF membranes are enriched, specifically with the growth factors released from the activated platelets that were initially stored in the granules [19,20]. One reason is based on the ability of certain growth factors and other bioactive molecules to bind to the PRF’s fibrin-rich matrix [21]. Thus, the PRF membrane serves as a pool of bioactive growth factors. Support for this statement comes from findings that the amount of growth factors is significantly higher in PRF membranes than in the corresponding PRF serum, except for IGF-1 [22]. Thus, activated platelets and the leucocytes entrapped in the PRF membrane release a myriad of bioactive molecules not restricted to those binding to the fibrin-rich matrix that should accumulate in the serum [23]. However, serum is not a relevant source of classic growth factors, further supporting findings that PRF membranes, but not PRF serum, are rich in growth factors such as EGF, PDGF-BB, TGF-β, and VEGF [22,24]. Moreover, growth factors accumulating within PRF membranes do not necessarily have to be released, as in vivo cells can migrate into PRF membranes via plasminogen, a system that is also required for inflammation resolution [25], including periodontitis [26]. Thus, migrating cells can liberate growth factors stored in the PRF membrane and, in turn, respond to their bioactivity. However, a cell response is complex and cannot be attributed solely to growth factors, as there are other bioactive molecules in PRF membranes and PRF serum; thus, there is a rationale for comparing the cell response of PRF membranes and PRF serum.

To establish a cell-based bioassay, we used gingival fibroblasts that had already served as a bioassay to study the activity to PRF membranes [27]. Single-cell transcriptomics of human oral mucosa have identified fibroblasts as a major source of chemokine–signaling molecules controlling the influx of neutrophils and other leukocytes in periodontitis patients [28]. However, the reason for their massively increased chemokine expression remains unknown. The inflammatory exudate containing cytokines and chemokines [29], similar to what we know from the gingival crevicular fluid [30], could drive this inflammatory fibroblastic cell response; however, we should not rule out that the activated platelets of coagulated plasma can also support fibroblast polarization towards an inflammatory phenotype. The response of gingival fibroblasts to PRF membranes and PRF serum further represents a clinical scenario where the local fibroblastic cells are exposed to a regular blood clot. The present research aims to identify the global response of gingival fibroblasts to PRF membranes and PRF serum based on bulk RNAseq analysis. This screening method allows us to differentially analyze the changes in the genetic signature of gingival fibroblasts, revealing a potential polarization switch.

## 2. Material and Methods

### 2.1. Isolation and Treatment of Gingival Fibroblasts

Explant cultures of gingiva from five healthy individuals who provided informed consent were used to isolate gingival fibroblasts. Ethical approval was provided by the Ethics Committee of the Medical University of Vienna (EK #631/2007). These cells were isolated and expanded in αMEM supplemented with 10% fetal calf serum (FCS) and 1% antibiotics (all Invitrogen Corporation, Carlsbad, CA, USA). A pool of the five donors of gingival fibroblasts, all at the first passage, were seeded at 30,000 cells/cm^2^ into 24-well plates and exposed to three individual 30% PRF membrane lysates and 10% PRF serum for 6 h in serum-free αMEM followed by isolation of the total RNA.

### 2.2. Preparation of PRF Membrane Lysates and PRF Serum

The preparation of PRF was approved by the Ethics Committee of the Medical University of Vienna (EK #1644/2018) and guided by the principles of the Helsinki Declaration and Good Clinical Practice. To prepare PRF membranes, blood from three healthy adult donors was centrifuged at 700× *g* for 8 min (swing-out rotor; Z306 Hermle, Universal Centrifuge, Wehingen, Germany) in glass tubes (Bio-PRF, Venice, FL, USA). The yellow PRF clot was separated from the remaining red thrombus. The PRF clot was compressed between two metal plates to generate solid PRF membranes and liquid PRF serum. The PRF serum was collected. One cm of PRF membrane was transferred to one mL serum-free αMEM and subjected to two cycles of freeze-thawing followed by sonication (Sonopuls 2000.2, Bandelin electronic, Berlin, Germany). The PRF membrane lysates were the supernatants obtained after centrifugation of the disintegrated PRF membranes at 15,000× *g* for 10 min (Eppendorf, Hamburg, Germany).

### 2.3. Total RNA Isolation, Sequencing and Data Analysis

Total RNA was isolated using the GeneMATRIX Universal RNA purification kit with DNAse digestion (EUR_X_, Gdańsk, Poland). Sequencing libraries from the total RNA were prepared at the Core Facility Genomics, Medical University of Vienna, using the QuantSeq 3′ FWD protocol version 2 with unique dual indices (Lexogen GmbH, Vienna, Austria). Fifteen PCR cycles were performed for library prep, as determined by qPCR according to the library prep manual. Libraries were QC-checked on a Bioanalyzer 2100 (Agilent Technologies, Santa Clara, CA, USA) using a high sensitivity DNA Kit for the correct insert size and quantified using a Qubit dsDNA HS Assay (Invitrogen, Waltham, MA, USA). Pooled libraries were sequenced using a P2 flowcell on a NextSeq2000 instrument (Illumina, San Diego, CA, USA) in 1 × 75 bp single-end sequencing mode. On average, 7 million reads per sample were generated. Reads in fastq format were generated using the Illumina bcl2fastq command line tool (v2.19.1.403) and the Lexogen idemux tool for optimal demultiplexing of long unique dual indices. Reads were trimmed and filtered using cutadapt version 2.8 to trim polyA tails, remove reads with N’s, and trim bases with a quality of less than 30 from the 3′ ends of the reads [31]. On average, 5 million reads were left after this procedure. Trimmed reads in fastq format were aligned to the human reference genome version GRCh38 with Gencode 29 annotations using STAR aligner [32] version 2.6.1a in 2-pass mode. Raw reads per gene were counted using STAR. Differential gene expression was calculated using DESeq2 [33] version 1.22.2.

### 2.4. Volcano Plot, Venn Diagram and Gene Set Enrichment Analysis

For volcano plot generation, we used VolcaNoseR, a web-based tool [34]. The up-and-down-regulated genes were used for further analysis under the premise of a minimum log2 with 2.5-fold change and a minus log10 significance level of two [34]. InteractiVenn, another web-based tool, was implemented to analyze gene sets through Venn diagrams [35]. The g:Profiler was used as a functional enrichment analysis tool that integrates many databases, including Gene Ontology and KEGG [36]. The sequence-based data are presented in GEO, the public functional genomics data repository, under the accession number GSE268248 released 31 May 2024.

## 3. Results

### 3.1. Principal Component Analysis and Heat Map of Gene Expression Changes by PRF Membrane Lysates and PRF Serum

**Figure 1:**
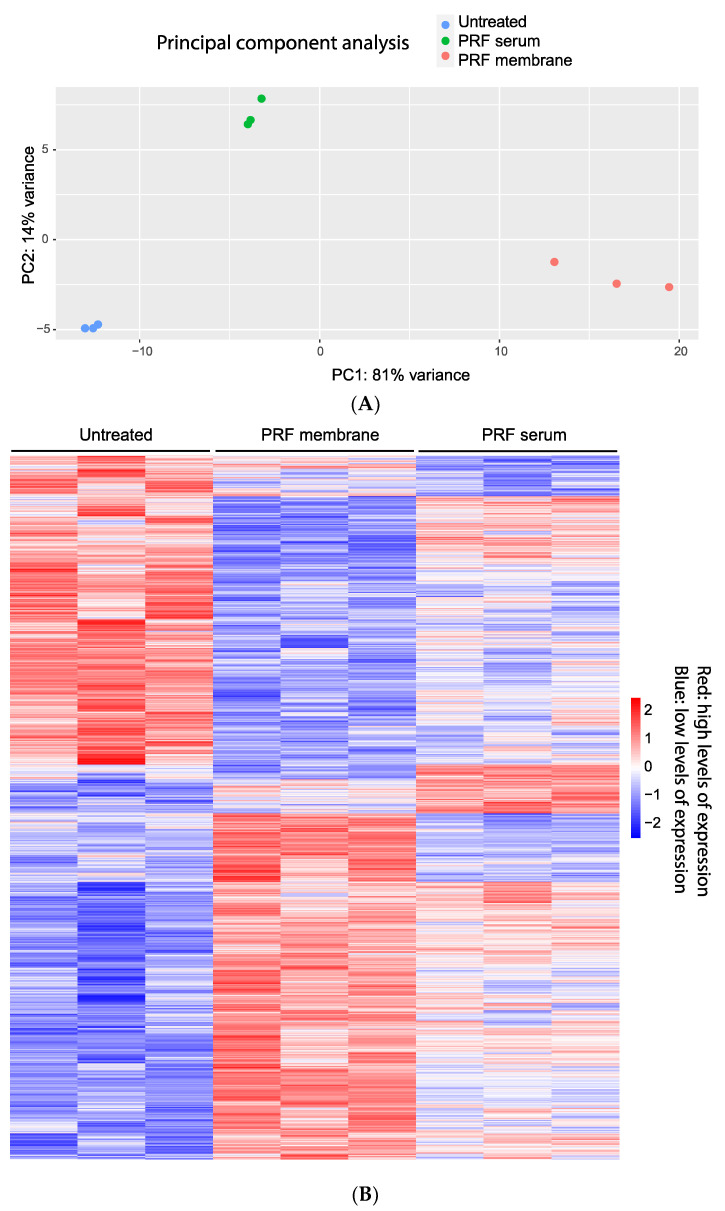
(**A**): Principal component analysis for differentially expressed genes in gingival fibroblasts treated with PRF membrane lysates and PRF serum. The analysis shows the homogeneity of individual donors in each treatment group and the heterogenicity caused by the treatment of the cells with PRF membrane lysates (PRF; red) and PRF serum (Serum, green). Untreated cells are indicated by the blue dots. (**B**): Heat map analysis for differentially expressed genes in gingival fibroblasts treated with PRF membranes and PRF serum. Each row represents a gene; the three columns represent different preparations of PRF membrane lysates and PRF serum. Red specifies high levels of expression, while blue shows low levels. Expression levels are indicated by darker and lighter shades of red and blue. Genes with a corrected *p*-value < 0.05 and an average log2 fold change ≥1 or ≤−1 were included in this analysis.

To investigate the degree to which PRF membrane lysates and PRF serum alter the transcriptional signature of human gingival fibroblasts, we conducted bulk RNA sequencing of gingival fibroblasts pooled from five donors and exposed to 30% PRF lysates and 10% PRF serum from three independent donors for 6 h. We have selected 10% PRF serum as higher concentrations lower cell viability. The principal component analysis reveals good reproducibility within the PRF donors, and the different treatments could be separated within the two dimensions (Figure 1A). We then performed a heatmap analysis to visualize the gene expression pattern of the PRF membrane lysates and PRF serum. The transcriptional heterogeneity between untreated and gingival fibroblasts exposed to the PRF lysates and PRF serum becomes evident in the heatmap based on the average expression of cluster-defining genes (Figure 1B).

### 3.2. Volcano Analysis of Gene Expression Changes by PRF Membrane Lysates and PRF Serum

**Figure 2:**
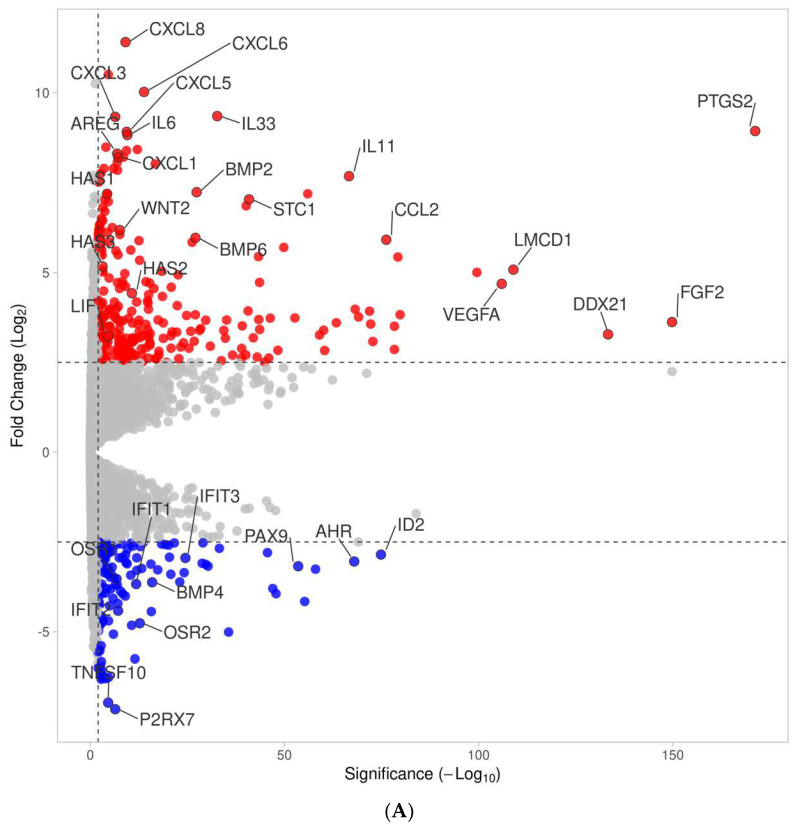

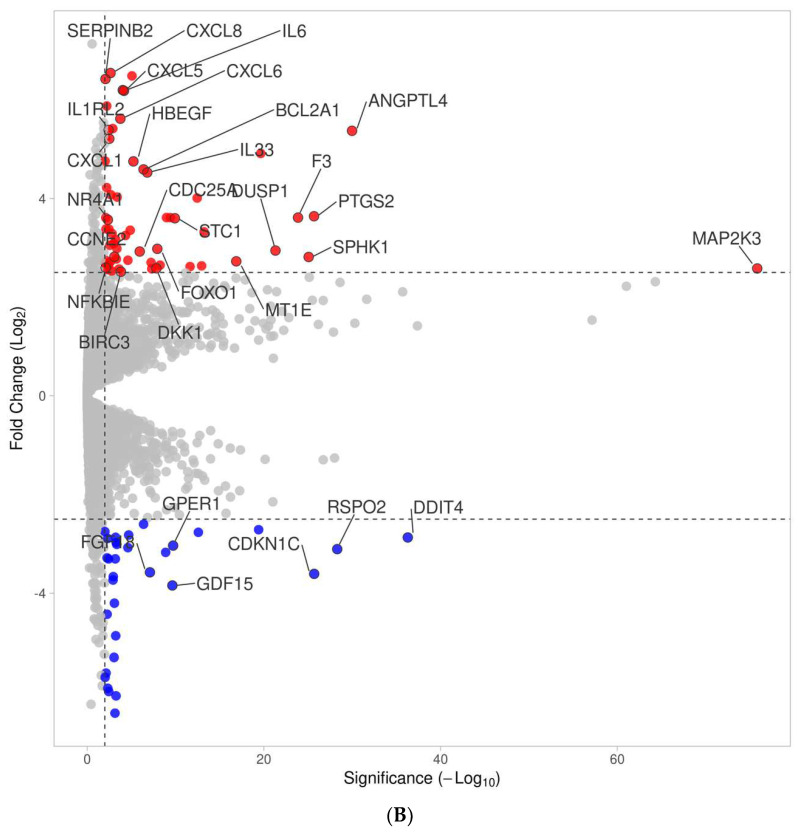
(**A**): Volcano plot analysis of differentially expressed genes in gingival fibroblasts treated with PRF membrane lysates. Volcano plot analysis identified up-regulated (red) and down-regulated (blue) genes in gingival fibroblasts treated with PRF membrane lysates. The annotated dots are data points at the largest (Manhattan) distance from the origin and are above the thresholds indicated by the dashed line. The threshold was set to at least a 2.5-fold change and a significance level of 2.0. (**B**): Volcano plot analysis of differentially expressed genes in gingival fibroblasts treated with *PRF serum*. Volcano plot analysis identified up-regulated (red) and down-regulated (blue) genes in gingival fibroblasts treated with PRF serum. The annotated dots are data points at the largest (Manhattan) distance from the origin and are above the thresholds indicated by the dashed line. The threshold was set to at least a 2.5-fold change and a significance level of 2.0.

We next performed a volcano analysis showing 268 and 136 genes up- and down-regulated using a PRF membrane lysates under the premise of at least log2 2.5-fold change and a minus log10 significance level of 2.0, respectively (Figure 2, Supplementary Table S1). In the case of the PRF serum, a volcano analysis revealed 62 and 32 genes that were up- and down-regulated under this premise (Figure 2, Supplementary Table S2). There were apparent similarities in the expression pattern of cells treated with the PRF lysates and PRF serum concerning chemokine expression, e.g., CXCL1, CXCL5, CXCL6, and CXCL8, but also IL33, IL6, PTGS2, and STC1. However, the response of gingival fibroblasts was more pronounced with the PRF membrane lysates than with the PRF serum, as exemplified by CCL2, CCL7, CXCL2, CXCL3, and IL1R1, IL1RL1, and IL1RN, as well as paracrine factors IL11, LIF, BMP2, FGF2, and HAS1, HAS2, and HAS3. Genes down-regulated by the PRF membrane lysates included IFIT1, IFIT2, IFIT3, and OSR1, OSR2; FGF18 and GDF15 were down-regulated by the PRF serum.

### 3.3. Venn Analysis of Genes Regulated by PRF Membrane Lysates and PRF Serum

Next, we performed a Venn analysis to display differences and similarities in the responses to PRF membrane lysates and PRF serum exposure. When comparing the genes commonly up- (Supplementary Table S3) and down-regulated (Supplementary Table S4) by PRF lysates and PRF serum, we identified 46 and 14 genes, respectively. Among the 46 genes were the chemokines CXCL1, CXCL5, CXCL6, and CXCL8, as well as DUSP1, F3, FOXO1, HBEGF, IL1RL2, IL33, IL6, NFKBIE, NR4A1, PTGS2, SERPINB2, SPHK1, and STC1. Less impressive were the 14 commonly down-regulated genes, including DBP and NR1D1.

**Figure 3:**
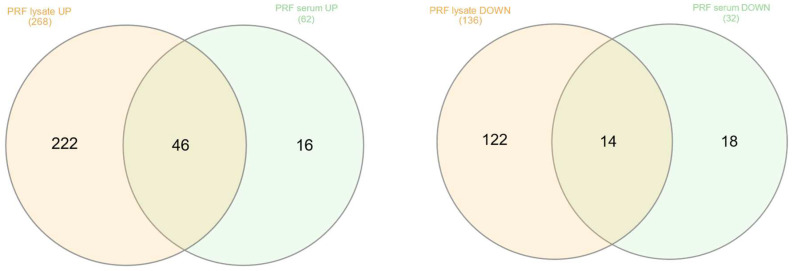
Venn analysis of the genes that were up- and down-regulated by PRF membrane lysates and PRF serum under the premise of at least 2.5-fold change and a significance level of two. A total of 46 and 14 genes were commonly up- and down-regulated by PRF membrane lysates and PRF serum, respectively.

This analysis further showed 222 and 16, as well as 122 and 18 genes that were independently increased and decreased by PRF membrane lysates and PRF serum, respectively (Figure 3). The genes significantly up-regulated by PRF lysates included the chemokines CCL2, CCL7, CXCL2, CXCL3, and IL1R1, IL1RL1, and IL1RN, as well as the paracrine factors IL11, LIF, IGF1, BMP2, BMP6, FGF2, CCN2/CTGF and HAS1, HAS2, HAS3. RT-PCR analysis confirmed the increased expression of CXCL1, CXCL2, CXCL8, CCL2, IL6, and IL33. The corresponding genes up-regulated by PRF serum included DKK1, CCNE2, and CDC25A.

Among the genes down-regulated exclusively by PRF membrane lysates were IFIT1, IFIT2, IFIT3, OSR1, and OSR2. The genes down-regulated by PRF serum included FGF18 and GDF15. Taken together, PRF membrane lysates, compared to PRF serum, cause a more complex response in gingival fibroblasts, with both including a noticeable increase in chemokine expression and other paracrine factors.

### 3.4. G:Profiler Analysis of Gene Expression Changes by PRF Membrane Lysates and PRF Serum

**Figure 4:**
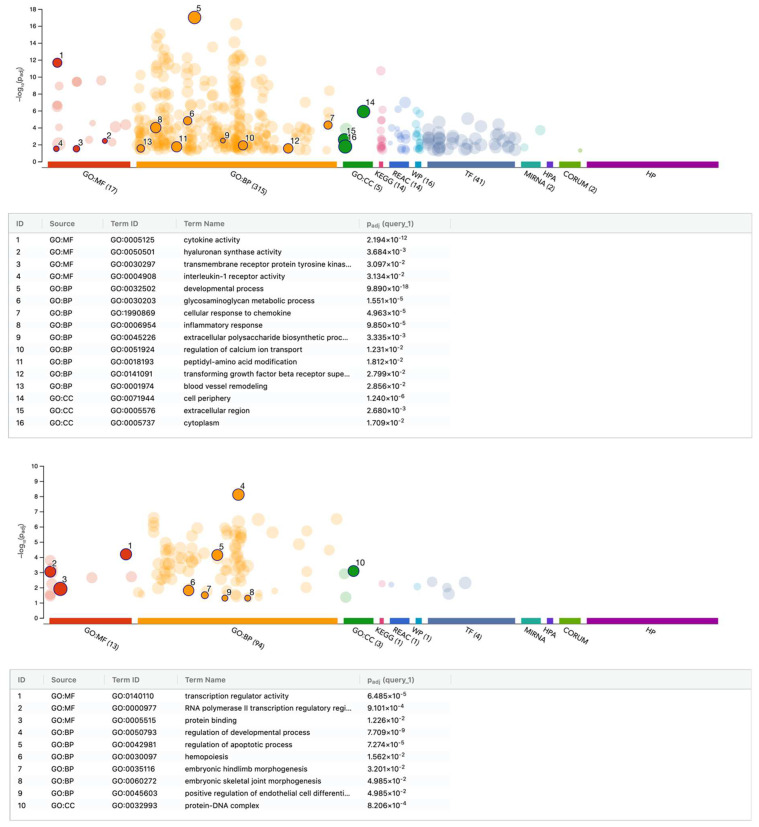
gProfiler analysis of differentially expressed genes in gingival fibroblasts treated with PRF membrane lysates. Functional enrichment analysis of genes that were (**upper**) up- and (**lower**) down-regulated by PRF membrane lysates. The enrichment analysis results are presented as a Manhattan plot, where the *x*-axis shows the functional terms grouped by the color code of the source database used. By contrast, the *y*-axis shows the enrichment-adjusted *p*-values in a negative decimal logarithmic scale. There are three types of terms listed in the gene ontology (GO): biological processes (BP), molecular functions (MF), cellular components (CC).

We further performed an enrichment analysis based on the differentially expressed genes in gingival fibroblasts exposed to PRF lysates (Figure 4). A summary of the analysis is reported in Supplementary Tables S5–S8. Gene ontology (GO) analysis for biological processes (BP), molecular functions (MF), cellular components (CC) was performed with all 268 up-regulated genes showing, for instance, GO:MF cytokine activity GO:0005125 with 24:238 genes: AREG, BMP2, BMP6, CCL2, CCL7, CLCF1, CXCL1, CXCL2, CXCL3, CXCL5, CXCL6, CXCL8, FGF2, GDF6, IL11, IL1RN, IL33, IL6, INHBA, INHBB, LIF, TGFB3, VEGFA, and WNT2. Also obvious was GO:MF hyaluronan synthase activity GO:0050501 with 3:4 genes: HAS1, HAS2, and HAS3. REAC signaling by TGFB family members identified 9:119 with BMP2, FSTL3, INHBA, INHBB, JUNB, MYC, PMEPA1, SMAD7, and TGFB3. Interestingly, MIRNA hsa-miR-98-5pis linked to 33:774 and MIRNA hsa-miR-223-3p are also linked to 9:99 genes, namely CXCL2, F3, FOXO1, IL6, LIF, PRDM1, SINHCAF, SLC7A5, and ZNF365 (Supplementary Table S5).

As indicated in the Supplementary Table S6, gene ontology of all 136 genes down-regulated by PRF membrane lysates revealed GO:MF transcription regulator activity GO:0140110 33:1952 with AHR, ALX1, ATOH8, BTG2, CITED2, DACH1, DBP, DYRK1B, EBF1, ETV2, FOXP2, FOXQ1, HHEX, MAF, MAMSTR, MXD3, NCOA7, NFE2, NR1D1, OSR1, OSR2, PAX9, RFXAP, RORB, RUNX1T1, SOX13, TEF, TRERF1, ZFHX4, ZHX2, ZNF135, ZNF510, and ZSCAN31.

**Figure 5:**
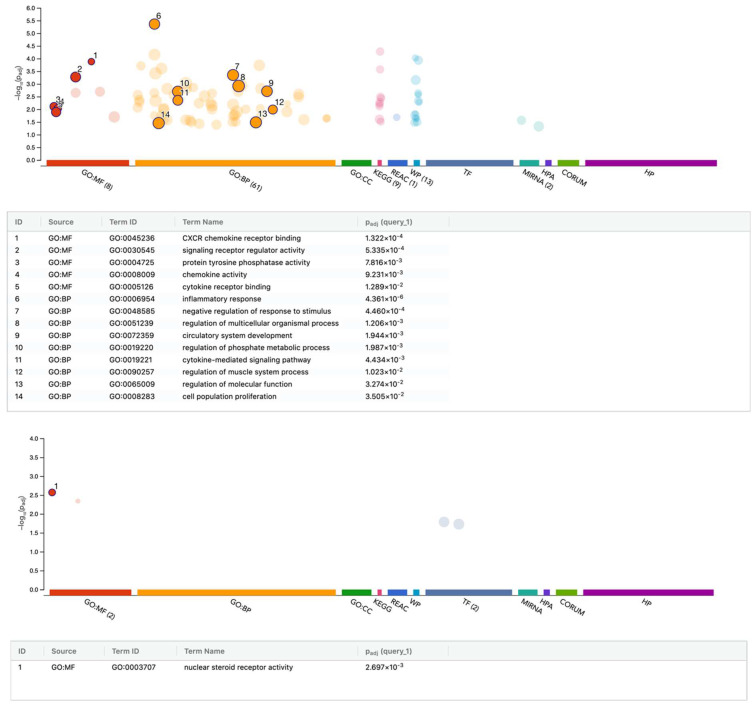
gProfiler analysis of differentially expressed genes in gingival fibroblasts treated with PRF serum. Functional enrichment analysis of genes that were (**upper**) up- and (**lower**) down-regulated by PRF serum. The enrichment analysis results are presented as a Manhattan plot, where the *x*-axis shows the functional terms grouped by the color code of the source database used. By contrast, the *y*-axis shows the enrichment-adjusted *p*-values in a negative decimal logarithmic scale. Three terms are listed in the gene ontology (GO) analysis: biological processes (BP), molecular functions (MF), cellular components (CC).

Furthermore, we performed an enrichment analysis based on the differentially regulated genes in gingival fibroblasts exposed to PRF serum (Figure 5). Gene ontology of up-regulated genes revealed GO:MF signaling receptor regulator activity GO:0030545 11:555 with ADM, CXCL1, CXCL5, CXCL6, CXCL8, DKK1, HBEGF, IL33, IL6, PDE4D, and STC1. Moreover, GO represents the gene ontology of down-regulated genes:MF nuclear steroid receptor activity GO:0003707 with 3:26 and GPER1, NR1D1, and PDE3A (Supplementary Tables S7 and S8).

## 4. Discussion

The local use of autologous PRF membranes has been widely established to support regeneration in dentistry [1,2,3,4,5,6,7,8] and other fields [9,10,11]. Thus, research to uncover the underlying molecular and cellular principles has become of increasing relevance, mainly when one fraction of coagulated plasma, the PRF membrane, is clinically used, while the other fraction, the PRF serum, is usually discarded. It is therefore important to know the activity of PRF membranes and what is lost to the PRF serum or PRF exudate [12,13,14]. The present research is relevant as we have compared the response of gingival fibroblasts serving as a bioassay to membrane lysates prepared from PRF membranes and the corresponding PRF serum. Our first main observation was that PRF membranes cause a more robust and more complex response than the PRF serum fraction, which brings us to the second observation, namely that PRF membrane lysates and the PRF serum cause a differential change in the signature of the gingival fibroblasts, suggesting unique activities, as well as similarities. Among the similarities is that the expression of a panel of chemokines is central to the migration of leucocytes in a hypothetical healing blastema, thus indicating potential clinical relevance of PRF treatment.

If we compare our results to those of others, we have to admit that a controversial observation was obtained using murine bioassays. Here, lysates from PRF membranes were potent in their ability to lower the forced expression of inflammatory cytokines and chemokines in, for instance, IL1β/TNFα-induced ST2 murine bone marrow mesenchymal cells [37] and LPS-exposed RAW 264.7 murine macrophages [38]. Thus, our initial conclusion was that PRF exhibits anti-inflammatory activity, but with the premise of a mouse-based bioassay [37]. Indeed, at this time, we were surprised that our preliminary data from gingival fibroblasts did not support observations with murine bioassays [37]. This has prompted us to switch to bioassays with human cells; consistent with our present observation, we were able to show that PRF membranes and, to a lesser extent PRF serum as well, fostered CXCL8 expression in gingival fibroblasts [39]. This observation was at the heart of our present RNAseq approach, in which we aimed to discover the global response of gingival fibroblasts to PRF membranes and PRF serum.

Based on RNAseq, we could now identify a panel of chemokines, including CXCL8 and cytokines IL6 and IL33, to be increasingly expressed in fibroblasts exposed to PRF membranes and, with lesser activity, to PRF serum. Apart from shedding light on the possible biological role of changing the paracrine activity of fibroblasts as observed in periodontitis [28], our observation highlights another aspect, namely, that the molecules responsible for the changes in chemokine expression are, at least partially, soluble and released into the serum—and are not necessarily being entrapped in the fibrin-rich matrix of the PRF membrane. The PRF membrane and the PRF serum likely use the same agonists to drive the expression of chemokines and other common genes, presumably similar to how normal blood serum forces human fibroblasts to increase inflammation genes such as cyclooxygenase-2, also known as prostaglandin-endoperoxide synthase 2 (PTGS2), as well as CXCL8, IL6, CCL2, CXCL12, CXCL2, ICAM1, and other serum responsive genes [40]. At this stage, we can only speculate which plasma- and serum-derived molecules account for the robust increase in chemokine and cytokine expression in the gingival fibroblasts.

One possible explanation would be that platelets or leucocytes release IL1 [41], and this cytokine is detected in human serum [42]. IL1 exhibits potent proinflammatory activity, enhancing chemokine and cytokine expression via gingival fibroblasts [43] or other commonly expressed genes, such as PTGS2 [44] and stanniocalcin-1 [45]. Moreover, in a situation of chronic IL1 exposure, resident cardiac fibroblasts can undergo a polarization switch to become a secretory cell for cytokines and chemokines [46], perhaps similar to what is observed with human preadipocytes [47]. Future research is therefore necessary to block IL1 signaling in gingival fibroblasts to see if the PRF membrane or PRF serum-induced gene expression changes are mediated via blood-derived IL1. Research is also required to understand the impact of lipids on the fibroblast response; for instance, we have shown that PRF lipids can mediate the effects of PRF on murine macrophages and stromal cells [48]. Thus, we have to investigate the impact of PRF lipids on the expression of chemokines and cytokines in gingival fibroblasts.

PRF membrane lysates can induce a gene expression profile in fibroblasts that is not achieved with PRF serum, as also indicated by the heat map analysis. Thus, growth factors and other bioactive molecules not being released into the PRF serum, or at least in insignificant amounts, are responsible for the signature change in PRF membrane lysates. One classic example is IL11, which is strongly induced by PRF membranes but not by PRF serum. Considering that IL11 is a major target gene of TGF-β [27,49,50] (the same is true for CCN2 [51], LIF [52], IGF1 [53], BMP2 [54], BMP6 [55], and FGF2 [56]), the present observation suggests that the majority of TGF-β activity remains in the fibrin-rich matrix and is not necessarily released into the PRF serum. Gene ontology further revealed that PRF membranes, but not PRF serum, regulate TGF-β signaling events. Moreover, our analysis disclosed another astonishing finding: PRF membranes increased the expression of all three isoforms of hyaluronic acid synthase (HAS). HAS expression could be related to the presence of TGF-β or IL1 in fibroblasts [57,58,59]. PRF membranes also increased a panel of IL1 receptors, namely, the IL1 receptor type I (IL1R1), also known as CD121a, the IL1 receptor antagonists (IL1RN), the IL1 receptor-like 1, also known as IL1RL1, and ST2, which presumably modulates IL1 sensitivity in cells. Moreover, PRF membranes increased chemokines CCL2, CCL7, CXCL2, and CXCL3, most of which are highly expressed in periodontitis fibroblasts [28]. Thus, our findings obtained with PRF membranes led us to ask the following: Which molecules, including TGF-β, are responsible for driving the signature change in periodontal fibroblasts?

The signature change includes genes being down-regulated by PRF membranes; these include IFIT proteins (interferon-induced proteins with tetratricopeptide repeats), which are supposed to mediate immunity against virus-related infection [60] and serve as biomarkers in oncology [61]. OSR1 and OSR2 (protein odd-skipped-related 1 and 2) zinc-finger transcription factors were likewise down-regulated. In mice, OSR2 is involved in the development of the palate and in suppressing the formation of teeth [62,63]. Conditional inactivation of OSR1 reduced myofiber growth and increased fibrotic tissue linked to TGFβ signaling, impairing overall muscle regeneration [64]. Down-regulated genes also involved ecotropic viral integration site 2A and EVI2B, as well as potassium voltage-gated channel subfamily B1 and S2 (KCNB1 and KCNS2), all of which exhibit unclear activity in periodontitis. Thus, the relevance of PRF membranes in lowering IFITs, OSRs, and other genes in gingival fibroblasts remains a matter of speculation.

In total, 16 genes were significantly up-regulated by the PRF serum but not the corresponding PRF membrane. For instance, PRF serum up-regulated genes such as CCNE2 and CDC25A linked to the cell cycle, as well as DKK1, a WNT-signaling antagonist with a potential impact on inflammatory osteolysis in rheumatoid arthritis [65] and in periodontitis [66]. Among the 32 genes down-regulated by PRF serum were FGF18, which facilitates tooth development by controlling periodontal tissue calcification [67] and bone regeneration in general [68]. Also, GDF15 is down-regulated; GDF15 is involved in osteoblast differentiation [69] and related to endocrinology [70]. Thus, attributing the effects of PRF serum to a specific signaling pathway or molecular function in gingival fibroblasts is challenging. PRF serum effects are perhaps more related to bioactive lipids accumulating in the serum more than in the PRF membrane [48]. However, further tests are required to understand if a lipid fraction of PRF, or even blood serum, causes the observed changes in expression, such as DKK1, FGF18, and GDF15. In general, current PRF research is almost exclusively limited to proteins, while insights into how blood lipids could exert a beneficial role in PRF membranes and serum are waiting to be discovered.

Technical limitations included the inability to standardize PRF membranes towards the PRF serum; we normalized lysates from PRF membranes based on the length of the membrane, which later underwent freezing and thawing [27,37,38]. Cells were exposed to a 30% PRF membrane lysates, knowing that this would exert robust activity in the form of the expression of IL11 and other genes [27,50]. With respect to the PRF serum, we were limited by its in vitro toxicity and established a 10% PRF serum to be appropriate for in vitro purposes. Theoretically, increasing the concentration could allow a PRF serum to exert a more robust response, but this was not feasible as the fibroblasts suffer. Dose–response experiments would presumably indicate a dilution ratio yielding a half-maximal effect of PRF on gene expression changes; this could perhaps be performed using a select panel of strongly regulated genes. Moreover, it is hard to standardize PRF in vitro; we prepared 1 cm of PRF membrane per 1 mL of medium, which was arbitrary but reproducible. Lysates of these “standardized” PRF membranes were diluted to a final concentration of 30%, but this did not strictly correlate with the 10% PRF serum. Although this may be reasonable if we assume that we obtain 1 cm of PRF membrane from 1 mL of PRF plasma, the calculation remains problematic. Another limitation was that our study was designed to have only one observation period of 6 h; thus, no long-term effects of PRF exposure could be observed. However, our short-time setting was intentional, as longer exposures provoke secondary effects caused by the autocrine effects of the expressed genes. Finally, another study limitation was that we used a lysates prepared from PRF membranes not being used in vivo. However, a PRF membrane, similar to a blood clot, undergoes fibrinolysis, a process that cannot be easily controlled or simulated in vitro. Thus, we chose freeze-thawing to prepare a lysates from the PRF membranes, as this procedure is fast, efficient and can be standardized.

Clinical implications remain a matter of speculation but also serve as a foundation for raising new hypotheses. For instance, if we assume that what we have observed in vitro also occurs under clinical conditions, we will have to explore how the changes in chemokine expression and other genes affect the early phase of regeneration, particularly concerning the application of PRF membranes. It is common knowledge that the early regeneration phase is inflammatory; however, the underlying molecular mechanisms need to be clarified. It can be hypothesized that, apart from the fast-released content of the platelets, molecules released from the blood clot or PRF, such as IL1, force the local fibroblasts to produce chemokines and other proteins that generate an inflammatory microenvironment, including the migration of neutrophils, monocytes, and lymphocytes; they may also produce hyaluronic acid and other extracellular matrix molecules. Future research is needed to extend our concept and compare a PRF membrane and serum to a regular blood clot [71]; likely, the response of fibroblasts to lysates prepared from a PRF membrane would be similar to that to lysates prepared from a blood clot, as the only variable would be the presence or absence of the erythrocytes [71]. Surprisingly, little is known about how a blood clot and the corresponding serum affect the genetic signature of fibroblasts and other cell types involved in tissue recovery.

In summary, the present research was based on a clinical scenario with the aim of differentiating between the response of PRF membranes versus PRF serum in a bioassay with gingival fibroblasts. Our research has led to a better understanding of the signature changes in an inflammatory phenotype that could exceed our original intentions, as it opens new approaches to how the early stages of wound healing and bone regeneration, as indicated by the formation of a blood clot, affect the microenvironment. Our overall findings suggest that PRF can provoke a polarization switch in fibroblasts toward a proinflammatory phenotype in vitro.

## Data Availability

All data are available on demand.

## Supplementary Materials

The following supporting information can be downloaded at: https://www.mdpi.com/article/10.3390/cells13151308/s1, Table S1: PRF regulated genes, Table S2: Serum regulated genes, Table S3: Upregulated genes Venn, Table S4: Downregulated genes Venn, Table S5: PRF upregulate GProfiler, Table S6: PRF downregulate GProfiler, Table S7: Serum upregulate GProfiler, Table S8: Serum downregulate GProfiler.
